# Energy metabolism and the intestinal barrier: implications for understanding and managing intestinal diseases

**DOI:** 10.3389/fmicb.2025.1515364

**Published:** 2025-01-31

**Authors:** Shuai Chen, Caifei Shen, Xiaorui Zeng, Luqiang Sun, Fangli Luo, Renhong Wan, Yupeng Zhang, Xinyun Chen, Yujun Hou, Wen Wang, Qianhua Zheng, Ying Li

**Affiliations:** ^1^Acupuncture and Tuina College, Chengdu University of Traditional Chinese Medicine, Chengdu, Sichuan, China; ^2^Hospital of Chengdu University of Traditional Chinese Medicine, Chengdu, Sichuan, China

**Keywords:** energy metabolism, intestinal barrier, SCFAs, cross-talk, intestinal diseases

## Abstract

The interplay between energy metabolism and the gut barrier is crucial for maintaining intestinal physiological homeostasis. Energy metabolism and the intestinal barrier perform distinct yet complementary roles that uphold intestinal ecological equilibrium. Disruptions in energy metabolism can compromise the integrity of the intestinal barrier; for example, inactivation of the AMPK pathway may lead to reduced expression of proteins associated with tight junctions. Conversely, impairment of the intestinal barrier can result in metabolic dysregulation, such as alterations in the gut microbiota that impede the production of short-chain fatty acids (SCFAs), which are essential substrates for energy metabolism. This disruption can affect energy production and modify the gut’s hypoxic environment. Imbalances in these systems have been associated with the onset of various intestinal diseases. Research indicates that dietary interventions, such as a low FODMAP diet, can enhance the colonization of probiotics and improve the fermentation metabolism of SCFAs. Pharmacological strategies to elevate SCFA levels can activate the AMPK pathway and rectify abnormalities in energy metabolism. This review provides a comprehensive summary of recent advancements in elucidating the interactions between energy metabolism and the intestinal barrier.

## 1 Introduction

The intestinal barrier and energy metabolism are essential physiological systems crucial for maintaining health. The intestinal barrier ensures environmental stability through the functions of the gut microbiota, the tight junctions between epithelial cells, and the local immune system. Energy metabolism encompasses the conversion and utilization of energy via biochemical reactions, which include energy acquisition, storage, and expenditure. This process is vital for sustaining life activities, supporting bodily functions, and ensuring overall health. Although at first glance there may appear to be no direct correlation between energy metabolism and the intestinal barrier, this is not the case. The gut microbiota plays a pivotal role in energy acquisition and storage, and alterations in its composition can significantly impact the body’s energy metabolism balance. Furthermore, disruptions in energy metabolism can compromise the integrity of the intestinal barrier, thereby increasing the risk of intestinal diseases. On the one hand, disruptions in energy metabolism can compromise the integrity of the intestinal barrier, increasing the risk of intestinal diseases. On the other hand, damage to intestinal barrier function may impair the effective utilization of energy. Disruptions in energy metabolism can compromise the integrity of the intestinal barrier, thereby elevating the risk of intestinal diseases. Conversely, impairment of intestinal barrier function may hinder the efficient utilization of energy. Recent research has increasingly elucidated the intricate interplay between the intestinal barrier and energy metabolism. Alterations in the gut microbiota can directly influence the host’s energy balance by modulating key pathways of host energy metabolism, such as the production of short-chain fatty acids and the metabolism of carbohydrates and lipids (Liu et al., 2018). Disruptions in energy metabolism have been demonstrated to affect the integrity of the intestinal barrier, leading to an increased incidence of leaky gut (Lin et al., 2022). These findings offer significant insights into the relationship between the intestinal barrier and energy metabolism; however, numerous questions remain unresolved. For instance, most studies have predominantly focused on unidirectional effects, thereby overlooking the dynamic relationship between the two, and the origins of this cross-talk remain unclear. This review seeks to explore the bidirectional interactions between the intestinal barrier and energy metabolism, as well as their implications for gut health and disease pathogenesis.

## 2 Intestinal barrier function

### 2.1 Mechanical barrier

The intestinal epithelial cell barrier plays a crucial role in protecting the organism from environmental challenges and maintaining epithelial integrity. This barrier undergoes a dynamic renewal process, with cellular turnover occurring every four to five days. The epithelial cell population is composed of various cell types, each serving distinct functions. Goblet cells, accounting for approximately 10% of the intestinal epithelial cells, primarily secrete mucus to safeguard the intestinal lining. Paneth cells are responsible for the production and secretion of antimicrobial peptides and proteins. Furthermore, exocrine and endocrine cells release a range of bioactive enzymes and cholecystokinin, respectively. Together, these diverse intestinal epithelial cells constitute the primary defense mechanism of the intestinal epithelial barrier (Gustafsson and Johansson, 2022). The mechanical barrier of the intestine is fundamentally based on the integrity of the mucosal epithelial cells, with particular emphasis on the tight junctions between them and the mucus layer that covers their surface (Lin et al., 2022). Disruption of the intestinal barrier facilitates the translocation of bacteria and antigens into the submucosa, which may result in the onset of gastrointestinal diseases (Simon et al., 2021). This compromise of the intestinal mechanical barrier constitutes a pathological mechanism that contributes to a range of gastrointestinal disorders, including irritable bowel syndrome (IBS) and inflammatory bowel disease (IBD) (Drossman, 2016; Edogawa et al., 2020; Ramos and Papadakis, 2019; Zhao L. et al., 2022).

### 2.2 Immune and chemical barrier

**FIGURE 1:**
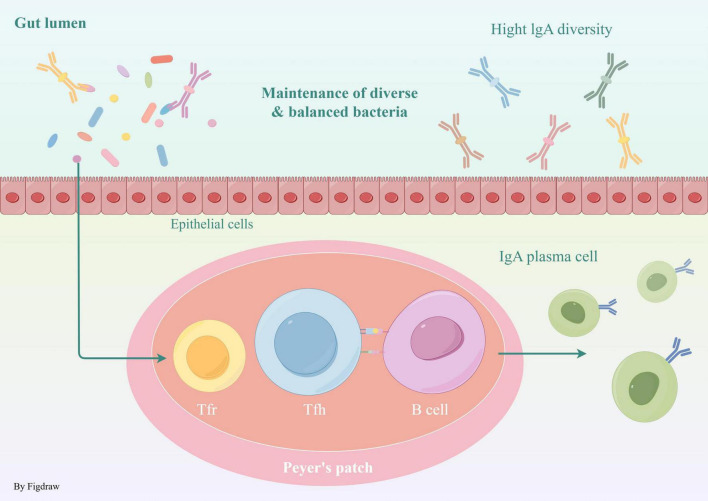
Biological barrier Tfh assist B cell proliferation and maturation, B cells produce IgA, which binds to pathogens and toxins, preventing their attachment to intestinal cells and neutralizing threats.

The chemical barrier primarily comprises mucus secreted by intestinal epithelial cells, digestive enzymes, and antimicrobial agents, including defensins and lysozyme, present within the intestinal lumen (Kalló et al., 2022). These substances possess the ability to directly eliminate or inhibit the proliferation of pathogens, thereby serving a crucial role in the prevention of infection and disease. Furthermore, these components are instrumental in modulating local immune responses and inflammatory processes, thereby enhancing the overall defense mechanisms against pathogenic threats (Chelakkot et al., 2018). The immune barrier encompasses gut-associated lymphoid tissue (GALT), which includes structures such as mesenteric lymph nodes and Kupffer cells located in the liver, as well as secretory immunoglobulin A (sIgA) produced by plasma cells within the gut. Additionally, mucosa-associated lymphoid tissue (MALT) contains a diverse array of immune cells, including T cells and B cells, which are adept at recognizing and responding to pathogens that infiltrate the gastrointestinal tract. Immune cells situated beneath the intestinal epithelial layer, such as dendritic cells and macrophages, are proficient in capturing and presenting pathogen-derived antigens, thereby initiating the activation of adaptive immune responses. Dendritic cells are capable of conveying pathogen-related information to other immune cells within the lymph nodes, thereby initiating a systemic immune response. In the gut, B cells predominantly secrete immunoglobulin A (IgA), which binds to pathogens and toxins, inhibiting their adhesion to intestinal epithelial cells (Reboldi et al., 2016). Follicular helper T (Tfh) cell growth is regulated by T follicular regulatory (Tfr) cells, providing assistance for B cell proliferation, selection, and affinity maturation (Linterman et al., 2011; Rescigno, 2014). This action effectively neutralizes harmful substances and prevents infection (Zhuang et al., 2024; Figure 1).

### 2.3 Biological barrier

The gut microbiota plays a pivotal role in energy metabolism and is composed of 12 distinct bacterial phyla, with the majority of gut bacteria classified under Proteobacteria, Firmicutes, Actinobacteria, and Bacteroidetes. These bacteria inhibit the proliferation of pathogenic species through mechanisms such as competitive exclusion, the production of organic acids, and the synthesis of antimicrobial substances. Additionally, they participate in intestinal metabolic activities, thereby contributing to the maintenance of the gut’s ecological equilibrium. In healthy adults, anaerobic Firmicutes and Bacteroidetes are the predominant bacterial groups within the gut (Hugon et al., 2015), The gut microbiota is capable of degrading undigested carbohydrates, leading to the production of SCFAs such as acetic, propionic, and butyric acid. These SCFAs serve not only as an energy source but also exert significant effects on gut health and overall metabolism.

## 3 Energy metabolism

Energy metabolism encompasses two primary types of reactions: anabolism and catabolism. Organisms derive energy by metabolizing fuel molecules, with the principal fuel molecules being: (1) glucose, which is primarily metabolized through glycolysis and the tricarboxylic acid (TCA) cycle; (2) fats, which undergo metabolism via fatty acid oxidation (β-oxidation); and (3) amino acids, which can be integrated into intermediates of glycolysis or the TCA cycle. The key processes involved in energy metabolism include glycolysis, the TCA cycle, oxidative phosphorylation, and fatty acid β-oxidation (Table 1 for details) (MacCannell and Roberts, 2022). Apart from glycolysis, mitochondrial function is essential for other metabolic processes. While glycolytic reactions occur rapidly and independently of mitochondrial activity, their ATP yield is comparatively low. Furthermore, under anaerobic conditions, glycolysis produces substantial quantities of lactate, resulting in an acidic microenvironment. This acidity can be detrimental to the survival and colonization of specific intestinal probiotics and may facilitate immune evasion in tumor cells (Zhao L. et al., 2022). Indeed, the interplay between the gut barrier and energy metabolism is critical, as energy metabolism significantly influences the integrity of the gut barrier, thereby underscoring a bidirectional relationship between these two systems.

**TABLE 1:** Energy metabolism process.

Way	Location	Process	Products
Glycolysis	Cytoplasm	Decompose one molecule of glucose (6 carbons) into two molecules of pyruvate (3 carbons each), producing 2 molecules of ATP and 2 molecules of NADH	Pyruvate, ATP, and NADH
TCA cycle	Mitochondrion	Acetoacetate is converted into acetyl CoA and enters the TCA cycle. Each molecule of acetyl CoA ultimately produces 3 molecules of NADH, 1 molecule of FADH_2_, 1 molecule of GTP (or ATP), and 2 molecules of CO_2_	NADH, FADH_2_, GTP (or ATP), CO_2_
OXPHOS	Mitochondrion	The high-energy electrons in NADH and FADH_2_ are transferred through an electron transfer chain, and the energy generated is used to combine ADP with inorganic phosphate (Pi) to generate ATP. The final electron combines with oxygen to form water	ATP and water
β-oxidation	Mitochondrion	Fatty acids are broken down into acetyl CoA through the β–oxidation cycle, while producing NADH and FADH_2_. Acetyl CoA subsequently enters the TCA cycle	Acetyl CoA, NADH, FADH_2_

## 4 Cross-talk between intestinal barrier and energy metabolism

### 4.1 The role of intestinal barrier function in energy metabolism

The intestinal barrier significantly contributes to energy metabolism through multiple mechanisms. First, it facilitates the absorption of essential fuel molecules, including glucose, fatty acids, and amino acids, which are fundamental to energy metabolism. Glucose is absorbed into the bloodstream via specific transport proteins, such as SGLT1 and GLUT2, located in the intestinal mucosal cells. Dietary fats are hydrolyzed into fatty acids and triglycerides, subsequently reassembled into chylomicrons for delivery to the liver for direct metabolism or entry into the bloodstream through the lymphatic system. Proteins are catabolized into amino acids, which are then transported into the bloodstream via specific transporters in the intestinal lining. Second, the intestinal barrier maintains the balance of the gut microbiota. The fermentation of dietary fiber by gut bacteria produces short-chain fatty acids, such as butyrate, propionate, and acetate, which supply energy to intestinal epithelial cells and contribute to the host’s overall energy homeostasis. Third, the intestinal barrier regulates metabolic signaling pathways. Incretins, such as GLP-1 and GIP, modulate blood glucose levels and metabolic equilibrium by influencing insulin secretion. Other hormones, including corticotropin-releasing hormone (CRH) and leptin, play a pivotal role in the regulation of metabolic processes and energy utilization through intricate signaling networks (Gao et al., 2023). Additionally, the regulation of inflammatory states is crucial, as the integrity and dysfunction of the intestinal barrier are linked to chronic inflammation, insulin resistance, and various metabolic disorders (Gomes et al., 2018; Zhang et al., 2020). Inflammatory mediators, such as TNF-α and IL-6, can elevate and disrupt insulin signaling pathways, thereby impacting energy metabolism (Bastard et al., 2006).

An imbalance in the gut microbiome reduces beneficial bacteria, increases harmful ones, or activates opportunistic pathogens, disrupting the production of SCFAs. These SCFAs are vital for energy, lipid and glucose metabolism, and appetite regulation. Their reduction can lead to obesity, insulin resistance, and metabolic disorders. A weakened chemical barrier increases gut inflammation risk. MyD88, an adaptor protein in inflammatory pathways, is crucial; mice without it in the gut are more susceptible to colitis and bacterial infections (Bhinder et al., 2014; Frantz et al., 2012). The targeted inhibition of the pro-inflammatory regulator NF-κB activation induces apoptosis in colonic epithelial cells, disrupts the expression of antimicrobial peptides, and facilitates bacterial translocation to the mucosa, consequently resulting in severe spontaneous intestinal inflammation in murine models (Nenci et al., 2007). Inflammatory mediators, such as TNF-α and IL-6, are pivotal in the pathogenesis of metabolic disorders, including obesity and type 2 diabetes mellitus. These cytokines disrupt insulin signaling pathways, culminating in insulin resistance, which profoundly impairs glucose metabolism, elevates blood glucose levels, and may precipitate metabolic syndrome. Compromise of the intestinal mechanical barrier, exemplified by the disruption of tight junction proteins, results in increased intestinal permeability. Consequently, macromolecules, including bacterial endotoxins and partially digested food particles, may translocate into the systemic circulation, eliciting a systemic inflammatory response. This inflammatory milieu can adversely affect systemic energy metabolism. Furthermore, impairment of the mechanical barrier may directly compromise nutrient absorption efficiency, leading to inadequate assimilation of glucose (Tamura et al., 2011), fatty acids and amino acids (Wada et al., 2013)—essential substrates for energy production—thereby resulting in energy deficits and potentially contributing to metabolic disorders (Groschwitz and Hogan, 2009). The interplay between energy metabolism and the integrity of the intestinal barrier is often reciprocal.

### 4.2 The impact of energy metabolism on the intestinal barrier

The complex and multifaceted impact of abnormal energy metabolism on the intestinal barrier involves intricate interactions among various systems, including the gut microbiota, intestinal epithelial cell function, and immune responses. Energy deficiency can compromise the integrity of the intestinal barrier, resulting in dysfunction, as intestinal epithelial cells require substantial ATP to maintain tight junction integrity, cell renewal, and repair processes (Lee et al., 2018; Lin et al., 2022). AMPK is a well-established central regulator of energy metabolism, activated in response to ATP deficiency. Upon activation, AMPK phosphorylates multiple downstream targets to stimulate ATP-generating catabolic pathways and inhibit ATP-consuming anabolic pathways. By inhibiting mechanistic target of rapamycin (mTOR) activity, AMPK regulates autophagy, thereby helping to restore energy balance and ensuring cell survival during energy stress (Herzig and Shaw, 2018). Adherens junctions (AJs) are particularly involved in activating AMPK in response to junctional tension. The activation of AMPK supports the barrier function of tight junctions (TJs) and promotes apicobasal cell polarization. This contributes to maintaining the integrity of the intestinal mechanical barrier, ensuring the proper functioning and structure of the intestinal epithelium (Bays et al., 2017; Lee et al., 2007; Rowart et al., 2018). This may be regulated by the AMPK mTOR pathway (Abdelmalak et al., 2023). Mitochondria serve as the “powerhouses” of organisms, playing a crucial role in ATP synthesis (Harrington et al., 2023), serving as the primary sites for maintaining redox homeostasis through the regulation of reactive oxygen species (ROS) production and clearance. Disruption of mitochondrial function can lead to an imbalance between ROS and the antioxidant system, thereby inducing oxidative stress (Wen et al., 2023). Elevated ROS levels have been implicated in adverse effects on human health, particularly in the context of neurodegenerative diseases and gastrointestinal disorders, as well as in the homeostasis of intestinal cells and the biodiversity of the gut microbiota (Ballard and Towarnicki, 2020). Furthermore, excessive ROS can impair mitochondrial function, diminishing the efficiency of the mitochondrial electron transport chain (ETC) and resulting in ROS accumulation and oxidative damage within the mitochondria (Ziegler et al., 2015). During oxidative stress, free radicals can alter the structure of intestinal tight junction proteins, leading to their disruption and decreased expression of occludin and ZO-1 proteins. This disruption compromises the intestinal mechanical barrier, increasing the risk of infections (Chen and Chou, 2016; Guttman and Finlay, 2009; Li et al., 2024). Moreover, impairment of the mechanical barrier enhances intestinal permeability, facilitating the infiltration of substantial quantities of deleterious substances. This process promotes the colonization of aerobic bacteria and results in dysbiosis of the gut microbiota (Reese et al., 2018). Excessive caloric intake can compromise the integrity of the intestinal barrier. A diet high in fat typically induces modifications in the gut microbiota, particularly disrupting the Firmicutes to Bacteroidetes ratio. This microbial imbalance, termed “dysbiosis,” undermines the function of the microbial barrier, further increasing intestinal permeability and facilitating the translocation of toxins, inflammatory mediators, and other harmful substances across the intestinal epithelium into the systemic circulation (Amabebe et al., 2020).

The metabolic processes of colonic epithelial cells predominantly utilize oxidative phosphorylation (OXPHOS) to achieve elevated oxygen consumption, thereby sustaining an anoxic environment within the intestinal lumen. This condition is conducive to a gut microbiota primarily consisting of anaerobic bacteria. Under physiological circumstances, SCFAs, which are metabolic byproducts of microbial activity, serve as critical signaling molecules for intestinal epithelial cells. Butyrate as an energy substrate for colon epithelial cells and modulates mitochondrial metabolism through multiple mechanisms. It facilitates the conversion of butyric acid to nicotinamide adenine dinucleotide (NADH) within mitochondria, thereby contributing to the OXPHOS process. Furthermore, butyric acid can enhance the expression of mitochondrial uncoupling protein 2 (UCP2), leading to increased proton leakage across the inner mitochondrial membrane and consequently diminishing the production of reactive oxygen species (ROS) (Gruber and Kennedy, 2017; Zhang et al., 2022). The depletion of microbial SCFAs as a result of antibiotic treatment may shift the oxygen metabolism of intestinal epithelial cells from OXPHOS to aerobic glycolysis. This metabolic shift leads to enhanced oxygenation of epithelial cells and facilitates the diffusion of oxygen into the intestinal lumen, thereby disrupting the anaerobic milieu of the gut (Byndloss et al., 2017). Moreover, the decline in butyrate-producing bacteria diminishes the mitochondrial activity of intestinal epithelial cells, resulting in the upregulation of the Nos2 gene, which encodes inducible nitric oxide synthase (iNOS). The upsurge in iNOS activity leads to increased production of nitric oxide (NO), which has the capacity to convert lactose and glucose into mucin acid and gluconic acid, respectively. This biochemical transformation fosters the proliferation of pathogenic bacteria such as Salmonella typhi. Additionally, nitric oxide can be metabolized into nitrate, which serves as a substrate for Vibrio parahaemolyticus, thereby exacerbating the intestinal inflammatory response (Faber et al., 2016; Liu et al., 2021). Opportunistic and pathogenic bacteria in the gut can induce mitochondrial dysfunction in intestinal epithelial cells through their toxins, resulting in elevated oxygen levels in the intestinal lumen. For example, Salmonella employs its virulence factors to recruit granulocytes to the intestinal epithelial cells, thereby initiating an inflammatory response. The inflammatory response augments the oxygenation of epithelial cells, resulting in the diffusion of surplus oxygen into the intestinal lumen. This modified environment suppresses the proliferation of *Clostridium difficile* and diminishes the concentration of metabolic products such as butyrate and other SCFAs, thereby further compromising the conditions essential for OXPHOS (Lopez et al., 2016).

Furthermore, impairment of mitochondrial synthesis function or disruption of oxidative phosphorylation in intestinal epithelial cells directly results in decreased cellular proliferation and impaired dietary fat absorption. Compromised mitochondrial function is associated with a significant reduction in the expression of intestinal epithelial stem cell markers Olfm4 and Lgr5 (Berger et al., 2016; Rodríguez-Colman et al., 2017). Nevertheless, the observed reduction in marker expression is not correlated with aberrant cell apoptosis. This suggests that while mitochondrial dysfunction influences the regulation of stem cell markers, it does not directly result in increased cell death within this context. The depletion of intestinal stem cells compromises the integrity of the intestinal epithelial barrier, as these cells are crucial for the routine regeneration and maintenance of the epithelium. Their depletion impairs the barrier’s ability to effectively repair or renew itself, leading to inevitable damage to its mechanical integrity. This disruption may increase susceptibility to infections and inflammation, as well as impair nutrient absorption. Additionally, mitochondrial dysfunction can impact the production of chylomicrons and the transmembrane transport of lipids. Dietary lipids ingested accumulate as lipid droplets within the intestinal epithelial cells instead of being transported to adjacent organs, potentially due to the disruption of the Golgi apparatus structure in these cells. This phenomenon occurs due to the inability of chylomicron precursors to be assembled into chylomicrons within the Golgi apparatus, resulting in significant lipid accumulation within intestinal epithelial cells (Moschandrea et al., 2024). This process may represent one of the underlying mechanisms contributing to metabolic diseases associated with aberrant energy metabolism.

## 5 Abnormal intestinal barrier and energy metabolism in intestinal diseases

### 5.1 Irritable bowel syndrome

In individuals diagnosed with IBS, the intestinal microbiota composition markedly differs from that of healthy controls. Specifically, patients with diarrhea-predominant IBS (IBS-D) exhibit a significant reduction in Firmicutes and an increase in Bacteroidetes (Zhuang et al., 2018). In patients with IBS, both the composition and activity of bifidobacteria in fecal and mucosal samples are comparatively diminished. Furthermore, notable alterations are evident in the microbial community structure associated with IBS, characterized by an enrichment of bacteria linked to intestinal inflammation, including *Enterobacteriaceae*, *Streptococcus*, *Clostridium difficile*, and *Shigella*. Notably, *Clostridium difficile*, *Clostridium* cluster XVIII, and *Gemella* are exhibit significant enrichment in patients with constipation-predominant IBS (IBS-C) (Sciavilla et al., 2021). Compared to healthy individuals, patients with IBS exhibit a reduced abundance of butyrate-producing bacteria. Furthermore, Methanobacteria levels are elevated in individuals with IBS-C, whereas these levels are diminished in those with IBS-D relative to healthy controls (Chong et al., 2019; Pozuelo et al., 2015). SCFAs are primarily produced by *Bacteroides*, *Bacteroidetes*, and *Clostridium* (Adak and Khan, 2019), as above, it can affect glucose and lipid metabolism.

**FIGURE 2:**
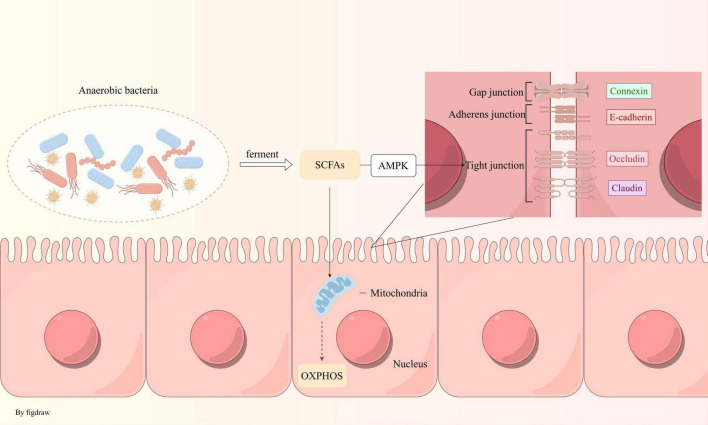
Anaerobic bacteria maintain an anaerobic environment in the intestine: anaerobic bacteria produce SCFAs via fermentation, which fuel oxidative phosphorylation in mitochondria, consuming oxygen to sustain intestinal hypoxia. These fatty acids also strengthen intestinal tight junctions through the AMPK pathway.

A clinical study has demonstrated that individuals with IBS-like symptoms exhibit more pronounced mitochondrial ultrastructural damage in duodenal epithelial cells compared to healthy controls. These IBS-like patients present with irregular mitochondrial morphologies, including swelling, flattening, and matrix cracking, accompanied by a reduction in matrix density (Miglietta et al., 2021). Alterations in mitochondrial structure can influence the composition of the gut microbiota. In a study involving mice with various mtDNA variants, a significant inverse correlation was observed between mitochondrial reactive oxygen species (ROS) production and microbial diversity. Mice with elevated ROS levels showed an increased abundance of *Bacteroidales* and a decreased presence of *Clostridiales* (Yardeni et al., 2019). This evidence suggests that IBS may be linked to energy metabolism disorders stemming from mitochondrial dysfunction, with potential bidirectional communication between mitochondria and gut microbiota. Dysbiosis of the gut microbiota leads to a decreased production of short-chain fatty acids (SCFAs), such as butyrate, which are essential for energy metabolism. This disruption results in reduced mitochondrial oxygen consumption, thereby altering the gut’s hypoxic environment and impeding the colonization of anaerobic bacteria (Figure 2). SCFAs levels further decline, exacerbating metabolic disorders and creating a vicious cycle. However, the initial origin of energy metabolism disorders in IBS remains unclear and requires further investigation.

Patients with IBS exhibit compromised intestinal integrity and disrupted tight junctions (Martínez et al., 2013; Piche et al., 2009), leading to altered intestinal permeability (Hanning et al., 2021). Although a comprehensive consensus has yet to be reached in IBS-related research, a reduction in colonic ATP levels has been documented in both human and animal studies (Chen et al., 2022; Zhang et al., 2019). Proteomic analyses suggest that the aberrant mechanisms of intestinal energy metabolism in IBS-D may be linked to specific associated proteins. ATP protein complexes, such as Atp5a1 and Atp5c1, play a crucial role in synthesizing ATP from ADP by utilizing transmembrane proton gradients to supply energy to the intestine (Liu et al., 2022).

The mucus layer overlaying the epithelial cells is synthesized by goblet cells and functions to lubricate and protect, thereby minimizing direct interactions between bacteria and the intestinal lining. In the colon, the inner mucus layer ensures that commensal microorganisms maintain a safe distance from the epithelial cells, while the mucin glycoproteins in the outer layer provide nutrients and binding sites for these microorganisms (Juge, 2022). Chronic diarrhea can compromise the integrity of the mucus layer in the colon, leading to aberrant mucin synthesis. The endoplasmic reticulum (ER), a pivotal site for the biosynthesis and folding of transmembrane proteins, secretory proteins, and lipids, is abundantly present in intestinal tissues (Liu et al., 2020). Enhancing intestinal mucus secretion and barrier protection can be achieved by activating autophagy and inhibiting endoplasmic reticulum stress, which may ameliorate the abnormal mucin synthesis associated with IBS-D (Zhao et al., 2024). Furthermore, mucin abnormalities may influence the colonization of microbiota in the IBS colon (Da Silva et al., 2015; Juge, 2022). We hypothesize that the comparatively low abundance of probiotics in the colon of individuals with IBS may be attributed not only to aberrant mucin synthesis but also to the interaction between mucin O-glycan counterparts and the specific recognition of bacterial surface components. In the context of IBS, the structural integrity of mucin O-glycans might be compromised, potentially disrupting this interaction and thereby contributing to the diminished presence of probiotics (Da Silva et al., 2014).

As previously discussed, disruptions in the microbiota can lead to abnormal levels of short-chain fatty acids (SCFAs), with each SCFA influencing intestinal barrier function through distinct mechanisms (Fu et al., 2023). Among these, butyric acid has emerged as a focal point of SCFA research due to its ability to enhance the expression of trefoil factor (TFF) (Leonel and Alvarez-Leite, 2012), which helps maintain and repair mucin-related peptides in the intestinal mucosa (Hamer et al., 2008). In addition, butyrate modulates the expression of tight junction proteins, thereby reducing paracellular permeability between cells (Ma et al., 2012). This effect may be mediated through the activation of the AMPK pathway, promoting tight junction reassembly and upregulating the levels of Claudin-1, Claudin-3, and Claudin-4 proteins, while also rectifying Claudin-1 distribution anomalies. Additionally, butyric acid has been shown to elevate intestinal ATP concentrations and improve ATP levels in colon tissue, exhibiting a dose-dependent effect (Li et al., 2022; Yan and Ajuwon, 2017). Although some studies have identified a “dose-response effect” of butyric acid, its beneficial impact on intestinal health remains significant (Deleu et al., 2021). Further investigation and research into the clinical application of butyrate for the treatment of IBS remain necessary. While acetate and propionic acid are known to activate barrier functions via claudin-4 and enhance cellular activity, their potential involvement in energy metabolism remains uncertain, as existing studies predominantly concentrate on their anti-inflammatory pathways (Facchin et al., 2024; Saleri et al., 2022).

The restoration or augmentation of gut microbiota can lead to significant improvements in the symptoms of irritable bowel syndrome (IBS). For instance, the oral administration of Escherichia coli capsules has been shown to enhance the diversity of gut microbiota, primarily comprising Bacteroidetes and Firmicutes, thereby alleviating abdominal pain, diarrhea, and associated anxiety in patients with IBS-D (Guo et al., 2021). Additionally, a diet low in fermentable oligosaccharides, disaccharides, monosaccharides, and polyols (FODMAP) has demonstrated particular benefits for IBS-D patients characterized by reduced microbiota diversity and relatively compromised nutritional states. The primary mechanism appears to involve alterations in microbiota-derived metabolites. After four weeks on a low FODMAP diet, the microbiome of these IBS patients tends to resemble that of healthy individuals (Conley et al., 2024), However, the underlying mechanisms remain unclear, as some studies have indicated that a short-term low FODMAP diet does not significantly impact the levels of short-chain fatty acids (SCFAs) in patients (Nordin et al., 2023).

### 5.2 Inflammatory bowel disease

IBD is categorized into two primary types: ulcerative colitis (UC) and Crohn’s disease (CD). Chronic intestinal inflammation in IBD leads to irreversible damage and degeneration of epithelial cells, resulting in increased intestinal permeability and facilitating immune cell infiltration. Elevated levels of ROS, combined with reduced antioxidant levels, contribute to the initiation and progression of IBD, thereby establishing a connection between ROS and IBD (Iborra et al., 2011). In patients with IBD, such as those diagnosed with CD, the intestinal mucosa is typically infiltrated by a substantial number of inflammatory cells, including neutrophils, macrophages, and lymphocytes (Ina et al., 1997; Naito et al., 2007). The pathogenesis of IBD is characterized by an imbalance between Th cells and regulatory T cells, with a particular emphasis on the dysfunction of regulatory T lymphocytes. Crohn’s disease is marked by Th1 cell-mediated inflammation, which results in the excessive production of cytokines such as IL-12, IL-17, and IL-23, whereas UC is associated with cytokines like IL-4, IL-5, IL-10, and IL-13, produced by Th2-type T cells (Uniken Venema et al., 2017). The active inflammatory processes are accompanied by the production and release of ROS from infiltrating immune cells. This release of ROS, along with other inflammatory markers in the mucosal inflammatory environment, leads to progressive cellular and molecular damage, culminating in further tissue destruction (Tian et al., 2017). ROS are linked to intestinal dysbiosis (Kunst et al., 2023). During inflammatory processes, the gut microbiota can directly generate ROS, leading to DNA damage in infected cells (Collins et al., 2011; Irrazábal et al., 2014). Following bacterial infection, the DNA repair system undergoes alterations due to bacterial regulation. Further research is required to elucidate the underlying mechanisms governing the interaction between gut microbiota composition and oxidative stress in the pathogenesis of IBD. Compared to healthy individuals, IBD patients exhibit a reduced population of bacteria with anti-inflammatory properties and an increased prevalence of pro-inflammatory bacteria (Frank et al., 2007), the most common alterations include a decrease in *Firmicutes* and an increase in *Proteobacteria* and *Bacteroidetes* (Chen et al., 2021). A decrease in SCFA-producing bacteria has been observed in individuals with IBD (Machiels et al., 2014), negatively impacting the growth and energy metabolism of intestinal epithelial cells. Goblet cells within the intestinal epithelium secrete a mucus layer that covers the surface, serving a vital function in mucosal defense and repair. Under normal physiological conditions, mucus-degrading bacteria decompose mucin, particularly MUC2, a secretory mucin synthesized by goblet cells, thereby releasing byproducts that can be utilized by other bacterial species (Sicard et al., 2017). In patients with IBD, there is a marked increase in the total abundance of mucus-degrading bacteria (Png et al., 2010), which exacerbates the breakdown of intestinal mucus and exposes epithelial cells to environmental factors. Additionally, the reduction in short-chain fatty acids (SCFAs) adversely affects the regulation of mucin glycosylation. This leads to the destabilization of mucin, further degradation of the mucosal layer, and thinning of the intestinal mucosa due to inflammation associated with IBD. A study demonstrated that supplementation with lactic acid bacteria significantly mitigated physiological damage in mice with colitis, reduced the severity of colon inflammation, decreased the production of inflammatory mediators, and preserved the structure and function of the intestinal epithelium. This protective effect is potentially linked to the upregulation of tight junction proteins, which helps maintain the integrity of the intestinal mechanical barrier and reduces intestinal permeability (Qian et al., 2024). Research indicates that the oral administration of Epigallocatechin-3-gallate significantly mitigates experimental colitis in mice by augmenting gut microbiota abundance and enhancing SCFA levels. This process exerts antioxidant and anti-inflammatory effects, thereby safeguarding the colon from damage (Wu et al., 2021). The role of SCFAs extends beyond the maintenance of epithelial tight junctions; they also regulate epithelial and luminal bacterial interactions through the production of antimicrobial peptides (AMPs), which serve as primary defense mechanisms against pathogens. Notably, the expression of AMPs such as RegIIIγ and β-defensins is markedly compromised in Gpr43 knockout mice, while butyrate has been shown to activate AMP production in cells (Zhao et al., 2018).

The mechanisms underlying the interaction between energy metabolism and the intestinal barrier in IBD exhibit partial similarities to those observed in IBS, yet they are not entirely congruent. In the context of colitis, the adaptive metabolic processes of colonic epithelial cells facilitate maximal ATP production through both oxidative phosphorylation and glycolysis, even in the face of diminished mitochondrial biogenesis. This metabolic adaptation is integral to the restoration of mitochondrial function during the repair of the colonic epithelium, which is essential for satisfying the heightened energy requirements and supporting the proliferation and differentiation of intestinal epithelial cells. Mitochondria are essential for energy needed in tissue repair and maintaining the intestinal barrier (Lan et al., 2023). IBD may affect energy metabolism, including β-oxidation, glycolysis, and the TCA cycle (Noh et al., 2024; Scoville et al., 2018), In CD patients, acylcarnitine production, crucial for transporting fatty acids into mitochondria for β-oxidation, is reduced. Compared to healthy individuals, β-hydroxybutyrate levels, derived from acetyl-CoA during fatty acid β-oxidation, are also significantly lower, indicating impaired fatty acid β-oxidation in CD. The active inflammation in IBD increases energy expenditure through protein breakdown, suggesting altered lipid metabolism. This energy imbalance may be central to CD’s pathogenesis. Improving mitochondrial function in intestinal cells can boost OXPHOS, lower intestinal oxygen, and correct dysbiosis in IBD (Kapur et al., 2024).

### 5.3 Colorectal cancer

The progression of colorectal cancer (CRC) is associated with mitochondrial alterations (Ren et al., 2023). In aerobic conditions, cancer cells preferentially utilize glycolysis for energy production. This metabolic shift from OXPHOS to glycolysis leads to the accumulation of lactic acid in the tumor microenvironment, resulting in a decreased intestinal pH and the establishment of a more acidic milieu. Such acidity not only facilitates cancer cell survival and proliferation (Hu et al., 2020), but also impairs immune cell function, thereby further promoting tumor progression.

The involvement of gut microbiota in the development of colorectal cancer (CRC) has been substantiated by numerous studies and is conceptualized through the “driver-passenger model.” In this model, “driver” pathogens are implicated in the initiation of colorectal tumorigenesis by producing toxins and compromising the protective mucus layer of the intestinal epithelium. Subsequently, the CRC microenvironment facilitates the proliferation of specific opportunistic “passenger” bacteria, which exacerbates dysbiosis, promotes excessive epithelial cell proliferation, and induces chronic inflammation, thereby contributing to the progression of CRC (Avril and DePaolo, 2021; Tjalsma et al., 2012). Peptostreptococcus, an anaerobic bacterium, adheres to the intestinal mucosa and utilizes its surface proteins to activate the PI3K-Akt signaling pathway, thereby inducing pro-inflammatory responses and facilitating cellular proliferation (Long et al., 2019). Furthermore, it has been observed that Anaerobic Peptostreptococcus activates Toll-like receptors 2 and 4 (TLR2 and TLR4), resulting in elevated intracellular reactive oxygen species (ROS) levels, which subsequently facilitate cell proliferation during the progression of colorectal cancer (CRC) (Tsoi et al., 2017). The pivotal role of short-chain fatty acids (SCFAs) in the eradication of colorectal malignancies has increasingly been acknowledged (Wang et al., 2019). Butyrate is capable of inhibiting colorectal cancer (CRC) proliferation through various mechanisms, such as the induction of autophagy-mediated β-catenin degradation (Garavaglia et al., 2022), the upregulation of TLR4 expression, and the activation of the MAPK and NF-κB pathways (Xiao et al., 2018), The antiproliferative effect of acetate may be attributed to its influence on mitochondrial metabolism; acetate has been shown to reduce the proliferation of tumor cell lines HT29 and HCT116 and to decrease glycolysis under normoxic conditions (Sahuri-Arisoylu et al., 2021). In tissue samples obtained from colorectal cancer (CRC) patients, a reduced abundance of SCFA-producing bacteria has been observed (Song et al., 2020), A diet high in fiber has been shown to modulate the gut microbiota and effectively prevent the onset of colon cancer in mice through a mechanism dependent on butyrate. Butyrate accumulates in cancerous colonic cells and acts as an inhibitor of histone deacetylases (HDAC), thereby suppressing cell proliferation and promoting apoptosis by inhibiting the Wnt/β-catenin signaling pathway. This process ultimately prevents the development of intestinal tumors in mouse models (Donohoe et al., 2014). Enhancing the abundance of SCFAs presents a promising therapeutic strategy for the treatment of CRC. Current interventions with potential include dietary modifications, lifestyle changes, probiotics, prebiotics, synbiotics, pharmacological treatments, and fecal microbiota transplantation (FMT).

## 6 Conclusion

Energy metabolism and the intestinal barrier are closely linked and essential for gut health. The intestinal barrier, made up of epithelial cells and tight junctions, blocks harmful substances while allowing nutrients and water through. Short-chain fatty acids (SCFAs) like butyrate and acetate, produced from dietary fiber fermentation by gut microbiota, supply energy to these cells, aiding their growth and repair. Metabolic processes like glycolysis and oxidative phosphorylation affect the barrier’s function by managing cellular energy and redox balance, which supports tight junction proteins. Research on energy metabolism and the gut barrier is extensive, but often overlooks related pathways and lacks a holistic view. Most microbiome studies focus on microbial changes, while metabolomics research targets metabolic disturbances, missing upstream and downstream interactions. Future studies should adopt a comprehensive perspective to determine whether dysregulated energy metabolism damages the gut barrier or vice versa, or if they interact in intestinal pathology. For instance, 5-HT impairs mitochondrial function by inhibiting the AMPK-PGC-1α axis, worsening stress-induced gut barrier disruption. The reactivation of the AMPK pathway by 5-HT receptors has been shown to restore mitochondrial function and repair the gut mechanical barrier. However, the interaction between energy metabolism and the gut barrier implicated in these processes remains insufficiently understood. Elucidating the underlying mechanisms of this interaction could offer valuable insights into the pathogenesis and progression of gut-related diseases, as well as inform the development of novel therapeutic agents. In summary, the connection between energy metabolism and the intestinal barrier is vital for gut health and preventing intestinal diseases. Understanding this link could aid in creating strategies to prevent and treat these disorders.
